# Nutritional quality improvement of soybean meal by *Bacillus velezensis* and *Lactobacillus plantarum* during two-stage solid- state fermentation

**DOI:** 10.1186/s13568-021-01184-x

**Published:** 2021-02-05

**Authors:** Long Chen, Zijian Zhao, Wei Yu, Lin Zheng, Lijia Li, Wei Gu, Haiyan Xu, Bingdong Wei, Xiaogang Yan

**Affiliations:** 1 Institute of Animal Nutrition and Feed, Jilin Academy of Agricultural Sciences, No. 186 Dong Xinghua Street, Gongzhuling, 136100 Jilin China; 2 Institute of Agro-food Technology, Jilin Academy of Agricultural Sciences, Changchun, 130033 China; 3 Shandong BaoLai-LeeLai Bioengineering Co. Ltd., Tai’an, 271000 Shandong People’s Republic of China

**Keywords:** *Bacillus velezensis*, Antinutritional factors, Soybean meal, *Lactobacillus plantarum*, Two-stage fermentation

## Abstract

*Bacillus velezensis* is widely used for agricultural biocontrol, due to its ability to enhance plant growth while suppressing the growth of microbial pathogens. However, there are few reports on its application in fermented feed. Here, a two-stage solid-state fermentation process using *Bacillus velezensis* followed by *Lactobacillus plantarum* was developed to degrade antinutritional factors (ANFs) and improve soybean meal (SBM) nutrition for animal feed. The process was evaluated for performance in degrading SBM antinutritional factors, dynamic changes in physicochemical characteristics, microorganisms and metabolites. After two-stage fermentation, degradation rates of glycinin and β-conglycinin contents reached 78.60% and 72.89%, respectively. The pH of fermented SBM (FSBM) decreased to 4.78 ± 0.04 and lactic acid content reached 183.38 ± 4.86 mmol/kg. NSP-degrading enzymes (Non-starch polysaccharide, NSPases) and protease were detected from the fermented product, which caused the changed microstructure of SBM. Compared to uninoculated SBM, FSBM exhibited increased proportions of crude protein (51.97 ± 0.44% vs. 47.28 ± 0.34%), Ca, total phosphorus (P), and trichloroacetic acid-soluble protein (11.79 ± 0.13% vs. 5.07 ± 0.06%). Additionally, cellulose and hemicellulose proportions declined by 22.10% and 39.15%, respectively. Total amino acid content increased by 5.05%, while the difference of AA content between the 24 h, 48 h and 72 h of fermentation was not significant (*P* > 0.05). Furthermore, FSBM also showed antibacterial activity against *Staphylococcus aureus* and *Escherichia coli*. These results demonstrated that two-stage SBM fermentation process based on *Bacillus velezensis* 157 and *Lactobacillus plantarum* BLCC2-0015 is an effective approach to reduce ANFs content and improve the quality of SBM feed.

## Key points

- *Bacillus velezensis* plays a key role during two-stage solid-state fermentation
- Fermentation with *B. velezensis* and *L. plantarum* improves SBM quality for feed use
- As a novel qualified presumption of safety (QPS) strain, *B. velezensis* will have a broad application prospect in food and feed industry.

## Introduction

Soybean meal (SBM) is the most common source of plant protein used by the food and feed industries. However, glycinin and β-conglycinin, the main antigenic proteins in SBM, comprise about 30% and 40% of total SBM protein, respectively (Maruyama et al. 2000). When consumed by young piglets, these proteins have been shown to induce hypersensitivity that leads to malabsorption syndrome, growth depression, and diarrhea (Shi et al. 2017a). Furthermore, non-starch polysaccharides (NSP), mainly consisting of cellulose, hemicellulose, and pectin, are also found in SBM and have been implicated as causative factors of intestinal disorders in weanling piglets (Shi et al. 2017b). Notably, the generation of fermented SBM (FSBM) by addition of microorganisms, such as *Rhizopus oligosporus*, *Aspergillus oryzae*, *Lactobacillus brevis*, or *Bacillus subtilis*, may improve SBM nutritional quality by removing antinutritional factors (ANFs) that are detrimental to livestock, while also enhancing bioavailability of nutritional components (Feng et al. 2007; Hong et al. 2004; Wang et al. 2020).

In recent years, *B. velezensis* has been newly reclassified taxonomically as a distinct species, as supported by results of numerous studies employing average nucleotide identity (ANI), DNA–DNA hybridization (DDH), and core-genome-based phylogenetic analyses that phylogenetically distinguish it from *Bacillus methylotrophicus*, *Bacillus amyloliquefaciens* subsp. *plantarum*, and *Bacillus oryzicola* (Dunlap et al. 2016). However, phylogenetic analysis has also revealed that *Bacillus velezensis*, *Bacillus amyloliquefaciens*, and *Bacillus siamensis* were clustered tightly together to form the “operational group *Bacillus amyloliquefaciens*” (Fan et al. 2017). This classification scheme is interesting in that it reflects the characteristics of *B. velezensis* that make this species widely applicable to biocontrol applications (Ye et al. 2018), such as commercial biofertilizers and biopesticides that currently employ strains SQR9 (Zhang et al. 2015), 9912D (Pan et al. 2017), LM2303 (Chen et al. 2018c), and FZB42 (Fan et al. 2018). Meanwhile, numerous recent studies and reports have supported use of *B. velezensis* in areas of biocontrol and plant growth promotion (Adeniji et al. 2019). In our previous study, *B. velezensis* 157 had been identified, characterized, and shown to exhibit broad spectrum antagonistic activity toward pathogenic microbes, while also exhibiting various needed lignocellulolytic activities. Meanwhile, SBM has been shown to serve as the most efficient solid-state fermentation (SSF) substrate for *B. velezensis* 157 (Chen et al. 2018b). Therefore, the ability of *B. velezensis* 157 to degrade ANFs and improve nutritional quality of SBM warrants further exploration, especially as part of a two-stage fermentation strategy combining both aerobic and anaerobic SSF (first stage, aerobic; second stage, anaerobic fermentation) to ultimately degrade ANFs and enhance SBM nutritional value. To date, only a few studies have already been reported for the processing of agro-industrial waste using pairs of organisms to optimize the quality of fermented feed, such as *Bacillus subtilis* and *Enterococcus faecium* (Shi et al. 2017a), *Bacillus subtilis* and *Saccharomyces cerevisiae* (Chen et al. 2009), and *Bacillus subtilis* and *Bacillus coagulans* (Yeh et al. 2018). However, applications involving feed fermentation by *B. velezensis* have not yet been reported and potential beneficial effects of fermentation on ANFs, dynamic changes in physicochemical characteristics, microorganisms and metabolites of SBM during a novel two-stage SSF process warrant further investigation. In addition, *B. velezensis* can be recommended for the QPS list with the qualification ‘absence of toxigenic potential and absence of aminoglycoside production ability, which has a broad application prospect in feed additive and fermentation feed industry (Koutsoumanis et al. 2020a, b).

In the present study, SBM was inoculated with *B. velezensis* 157 during the first fermentation stage to remove ANFs from SBM. Next, the addition of *L. plantarum* BLCC2-0015, which has greater acidic capacity than *B. velezensis*, to induce second stage fermentation promoted lactic acid production and reduced the pH of inoculated SBM. During this two-stage fermentative process, microorganisms and metabolites, ANFs, and chemical composition profiles of uninoculated versus inoculated SBM were characterized and compared.

## Materials and methods

### Microorganisms and basal substrate

*Bacillus velezensis* 157 had been isolated from *Eucommia ulmoides* bark and was shown to exhibit various lignocellulolytic activities suited to SSF conversion of agro-industrial waste, which was collected in China Center for Type Culture Collection (M2017475). *L. plantarum* BLCC2-0015 (CTCC number M2015126) was obtained from Baolai-leelai Bio-tech Co., Ltd. (Taian, China) and is a widely accepted feed additive (originally isolated from pickles) that is used in mixed corn-soybean meal feed. In addition, *Staphylococcus aureus* ATCC25923 and *Escherichia coli* ATCC25922 were used as indicator organisms provided by the Institute of Animal Nutrition and Feed, Jilin Academy of Agricultural Sciences, China. Dried and corticate soybean meal (purchased from a local market in Gongzhuling, China) was sieved through a 40-mesh sieve prior to SSF.

### Protein degradation capacity

The protein degradation capacity of *B. velezensis* 157 and *L. plantarum* BLCC2-0015 were measured using the agar well diffusion method. The crude supernatant of candidate strains were prepared according to the method (Wongputtisin et al. 2012). Subsequently, the supernatants were determined using a soybean antigenic protein screening plate described by Liu. et al. (2007).

### Preparation of fermented soybean meal

Two-stage fermentation of feed was performed using SBM as substrate, which was sterilized at 121 °C for 20 min. Prior to fermentation, *B. velezensis* 157 was cultured for 12 h in liquid LB medium at 37 °C. *L. plantarum* BLCC2-0015 was cultured for 16 h in liquid MRS medium, at 37 °C. The vegetative cells were resuspended in sterile 0.85% NaCl (10^8^ CFU/mL) after washed thrice with sterile 0.85% NaCl.

The sterilized SBM (150 g) was conducted in a 500 mL Erlenmeyer flask with a sterile membrane, then sterile water and bacterial suspension in PBS to achieve a final moisture content of 40% in dry basis. During the first stage of fermentation, SBM was inoculated with *B. velezensis* 157 (8.0 log CFU/g) then fermentation was allowed to proceed for 24 h at 37 °C. For the second stage of fermentation, Erlenmeyer flask was sealed with a sterile rubber plug, the fermented mixture was inoculated with 8.0 log CFU/g of *L. plantarum* BLCC2-0015 then incubated for 48 h under anaerobic conditions at 37 °C. The addition of sterile 0.85% NaCl instead of inoculated bacteria were served as controls. All control and inoculated samples were tested in triplicate. 2 g of moist samples at 0 h, 24 h, 48 h and 72 h were collected for immediate determining for pH, microbial, enzyme activity, antimicrobial activity and lactic acid analyses, and the remaining samples were prevented continuous fermentation at 105 °C for 30 min (Shi et al. 2017a). Afterwards, all samples were dried for 24 h at 65 °C, cooled, ground, and subjected to SDS-PAGE, and physicochemical analysis.

### Microorganisms and metabolites

The pH and microbiological counts were analyzed as described by Jin et al. (2017). A lactic acid enzymology assay kit (Nanjing Jiancheng Technology Co., Ltd.) was used to determine lactic acid content using the manufacturer’s protocol provided with the kit. Cellulase, xylanase, pectinase and β-mannase activities were analyzed by DNS method (Blibech et al. 2020; Parab et al. 2017; Salim et al. 2017). The activity of neutral protease was detected as mentioned by Salim et al. (2017). Antimicrobial activity of FSBM was analyzed using an agar-well diffusion assay. The *Staphylococcus aureus* ATCC25923 and *Escherichia coli* ATCC25922 were used as indicator organisms for antimicrobial test (Su et al. 2018). Moist samples after two-stage fermentation were diluted in 0.85% NaCl and transferred into a well in the MH agar containing corresponding pathogens at 37 °C for 24 h. The control discs were impregnated with supernatant of uninoculated SBM.

### Chemical analysis

Dried samples at 0 h, 24 h, 48 h, and 72 h were analyzed according to AOAC International Guidelines (2005) to determine contents of dry matter (DM), crude fibre (CF), crude protein (CP), neutral detergent fiber (NDF), and acid detergent fiber (ADF), total phosphorus (P), Calcium (Ca), ash. The method reported by Ovissipour et al. was used to determine sample TCA-SP content (Ovissipour et al. 2013). An automated amino acid analyzer (LA8080; Hitachi, Tokyo, Japan) was used to determine amino acid profiles. Before analysis, the dried samples were hydrolyzed with 6 mol/L HCl at 110 °C for 24 h. Analyses of glycinin and β-conglycinin contents in uninoculated SBM and FSBM were conducted using an indirect competitive enzyme-linked immunosorbent assay (ELISA) kit as per kit instructions (Long zhou fang ke Bio Co., Ltd.).

### SDS-PAGE

A Plant Protein Extraction Kit (Beijing Solarbio Science and Technology Co., Ltd.) was used to extract soluble proteins from uninoculated SBM and FSBM according to the protocol provided. Next, sample protein concentrations were determined using a Bio-Rad Protein Assay Kit (Bio-Rad, USA) and an SDS-PAGE system utilizing 12% polyacrylamide separating gels for 120 min at 65 mV to fractionate soluble proteins, as reported by Shi et al. (2017a). Post-electrophoresis, gels were stained for 45 min with Coomassie Brilliant Blue R-250 (Bio-Rad, USA) then destained in 7% acetic acid.

### Microscopic inspection

Physical property changes of samples were examined by SEM according to the protocol of the Electronic Microscopy Center of Xi’an Lianyi Sharing Information Technology Co. ltd. The microstructures of uninoculated SBM and FSBM were observed using a field-emission SEM (JSM-7900, JEOL, Japan) at ×1000, ×1500, ×3000 and ×5000 magnifications.

### Statistical analysis

Data were processed and evaluated by Student’s t tests and one-way analysis of variance (ANOVA) with Duncan’s multiple-range test using SPSS software (SPSS Inc., Chicago, IL, USA). A P-value ˂ 0.05 indicated a significant difference between groups, with each result expressed as mean ± standard deviation. Histograms and line graphs were drawn using GraphPad Prism 8.0 software.

## Results

### Protein degradation capacity

As shown in Additional file 1: Figure S1 (a and c), *B. velezensis* 157 showed larger hydrolysis diameters on the soybean antigenic protein screening plate, while *L. plantarum* BLCC2-0015 does not affect.

### Microorganisms, pH, antimicrobial activity and lactic acid concentration during SSF

**Fig. 1:**
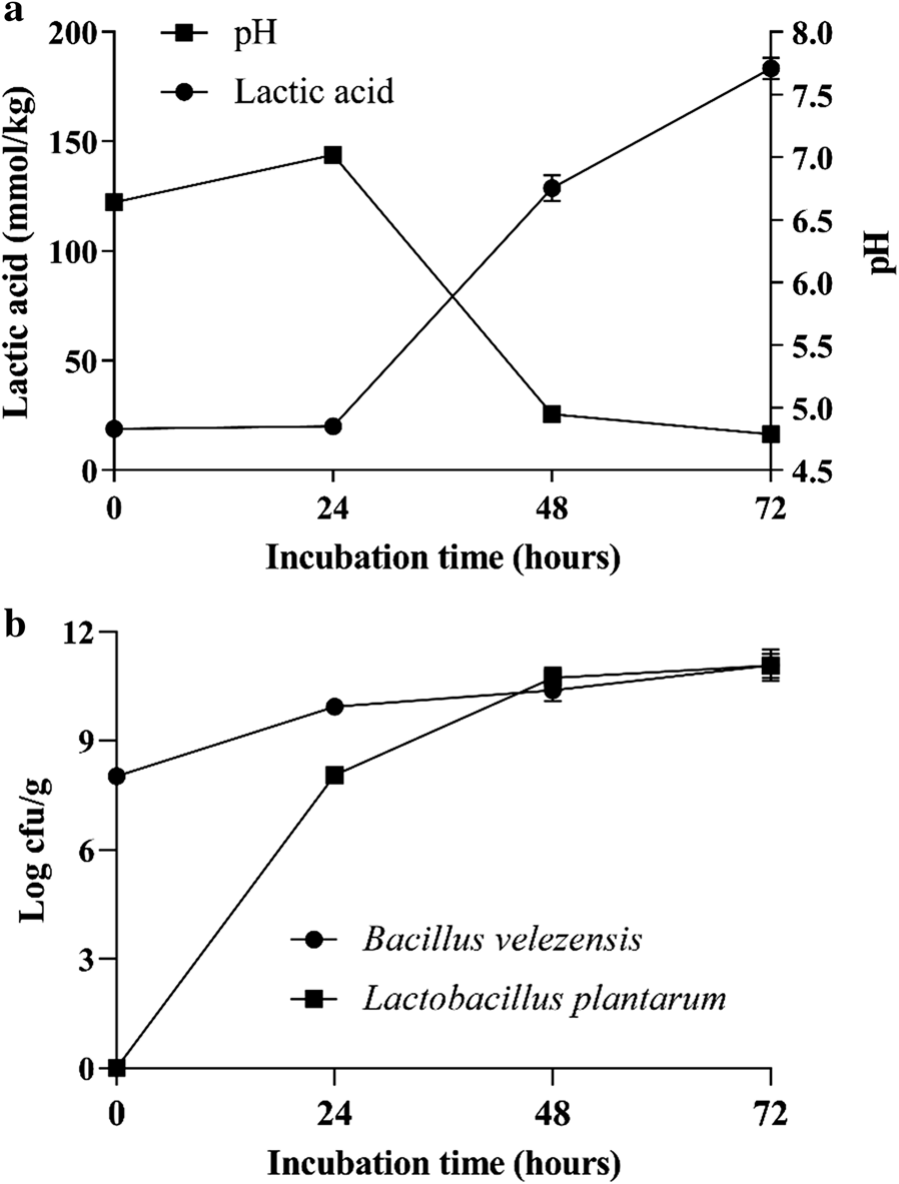
pH, lactic acid concentration (mmol/kg) (**a**) and the number of microbial (**b**) in uninoculated SBM and FSBM during two-stage fermentation

Two-stage fermentation based on activities of *B. velezensis* 157 and *L. plantarum* BLCC2-0015 significantly altered microorganisms, pH and lactic acid concentration of SBM (Fig. 1a). The initial density of *B. velezensis* 157 was 8.03 ± 0.05 log CFU/g. After 24 h of incubation, the density increased to 9.93 ± 0.15 log CFU/g. However, the number of *B. velezensis* 157 increase continuously under the second stage of anaerobic fermentation, and the final count was 10.38 ± 0.29 log CFU/g and 11.07 ± 0.43 log CFU/g at 48 h and 72 h of incubation. The density of *L. plantarum* BLCC2-0015 at 24 h was 8.05 ± 0.09 log CFU/g after inoculation, then the number of *L. plantarum* BLCC2-0015 increased to 10.72 ± 0.28 log CFU/g and 11.07 ± 0.33 log CFU/g at 48 h and 72 h in FSBM, respectively. During the anaerobic fermentation period, the number of *L. plantarum* BLCC2-0015 was similar to that of *B. velezensis* 157 (Fig. 1b). During the first stage of fermentation, almost no change in lactic acid content was observed, while a small increase in pH from 6.64 ± 0.02 to 7.02 ± 0.05 was observed post-incubation with *B. velezensis* 157. During the second-stage fermentation after *L. plantarum* BLCC2-0015 inoculated for 48 h, the pH gradually decreased from 7.02 ± 0.05 to 4.78 ± 0.04 and was accompanied by a marked increase in lactic acid content from 20.12 ± 2.31 to 183.38 ± 4.86 mmol/kg (Fig. 1a). Besides, FSBM harvested from 24 h, 48 h and 72 h of two-stage SSF showed antimicrobial activity against *S. aureus* (Additional file 1: Fig. S2 A-C) and *E.coli* (Additional file 1: Fig. S2 D-F) compared with uninoculated SBM.

### Enzymatic activities

**Fig. 2:**
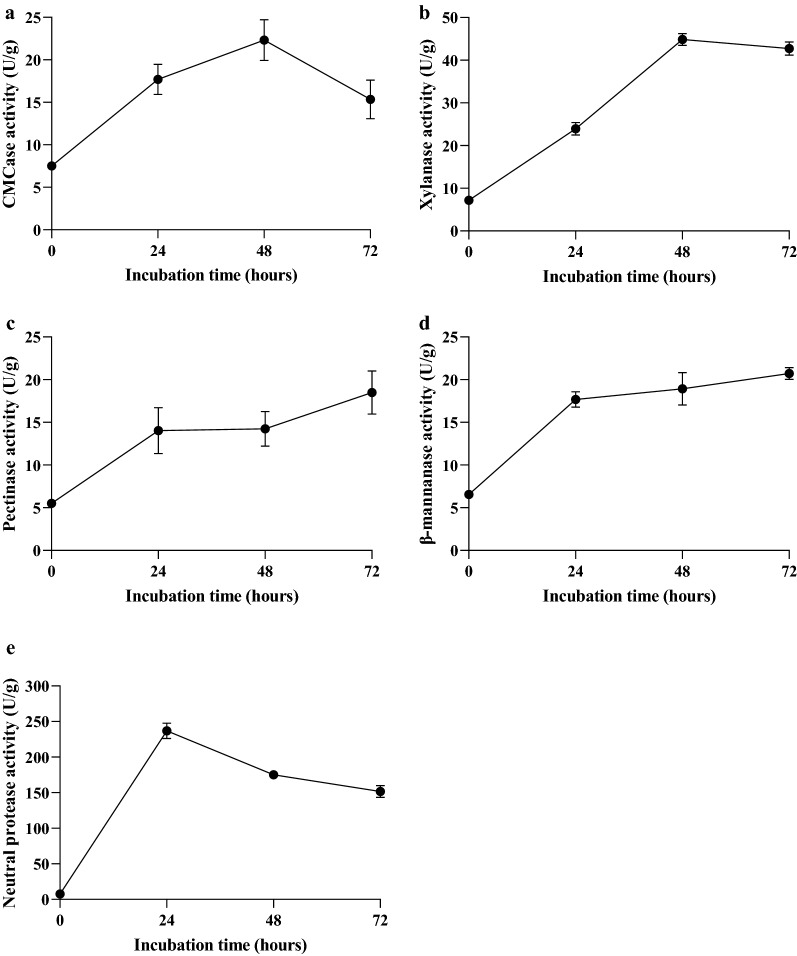
Production of cellulase (**a**), xylanase (**b**), pectinase (**c**), β-mannanase (**d**) and neutral protease (**e**) of uninoculated SBM and FSBM during two-stage fermentation

Production of cellulase, xylanase, pectinase, β-mannanase and neutral protease by two-stage fermentation were tested. Figure 2e showed that the maximum production of neutral protease was obtained by *B. velezensis* 157 (236.94 ± 10.75 U/g) during the first-stage fermentation, then decreased gradually with the inoculation of *L. plantarum* BLCC2-0015. Cellulase and xylanase exhibited their maximum activities 22.31 ± 2.41 and 44.72 ± 1.41 U/g at 48 h and thereafter the enzyme synthesis decrease (Fig. 2a, b). Initially, pectinase and β-mannanase productions were 14.24 ± 2.70 and 17.67 ± 0.89 U/g at 24 h and after that the enzyme activity remained constant. The highest pectinase and β-mannanase activity were found after 72 h of two-stage fermentation which were 18.49 ± 2.52 and 20.72 ± 0.69 U/g (Fig. 2c, d).

### Soybean antigenic protein biodegradation after two-stage fermentation

**Fig. 3:**
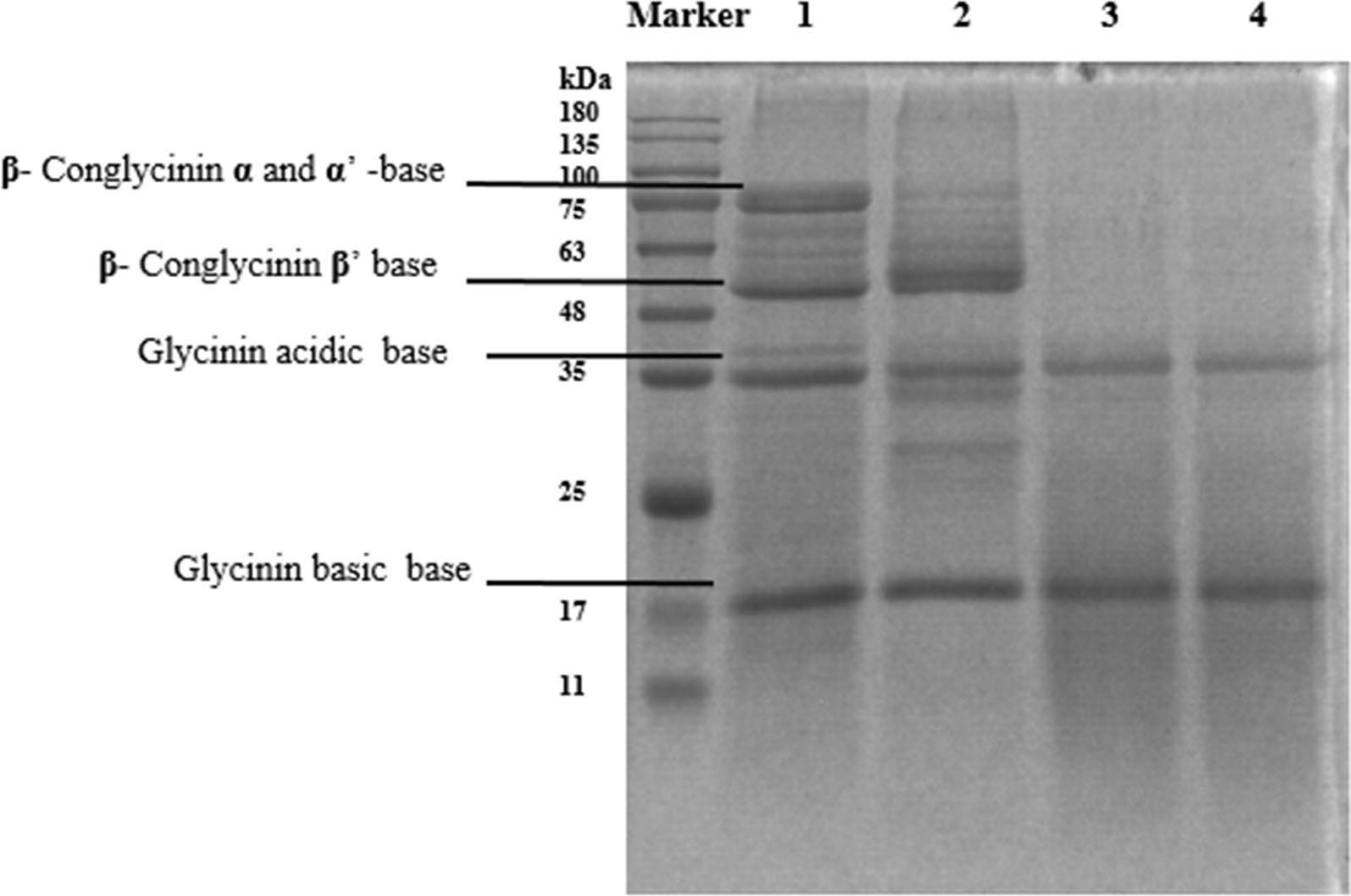
Effect of degrading glycinin and β-conglycinin of uninoculated SBM and FSBM during two-stage fermentation. Marker: protein molecular weight markers (11–180 kDa); 1: uninoculated SBM; 2: the SBM was inoculated with *B. velezensis* 157 and fermented at 37 °C for 24 h; 3: the first-stage fermentation was inoculated with *B. velezensis* 157, incubated at 37 °C for 24 h, then were inoculated with *L. plantarum* BLCC2-0015 and fermented under anaerobic conditions at 37 °C for another 24 h; 4:the first-stage fermentation was inoculated with *B. velezensis* 157, incubated at 37 °C for 24 h, then were inoculated with *L. plantarum* BLCC2-0015 and fermented under anaerobic conditions at 37 °C for another 48 h

The SBM yielded separate classic antigenic protein profile bands corresponding to soybean antigenic protein subunits in the range of 20 to 100 kDa, including β-conglycinin peptide subunits (α, α′, and β) and acidic and basic glycinin peptides normally found in SBM. After 24 h of fermentation, the antigenic protein of glycinin and β-conglycinin in FSBM were fractionally degraded into molecules with smaller masses (Fig. 3). Degradation rates of glycinin and β-conglycinin contents reached 68.14% and 66.41%, respectively, by 24 h of *B. velezensis* 157-induced fermentation. Interestingly, a further degree of antigenic protein degradation was observed during second-stage fermentation than during first-stage fermentation. Degradation rates of glycinin and β-conglycinin contents reached 76.62% and 71.77%, respectively, by 48 h of anaerobic fermentation. Respective degradation rates of glycinin and β-conglycinin contents further increased to 78.60% and 72.89% by 72 h of anaerobic fermentation, and the difference between the 48 h and 72 h of anaerobic fermentation was not significant (*P* > 0.05) (Table 1).

**Table 1:** Effect of fermentation on the concentration of soybean antigenic protein during two-stage fermentation

Item	Glycinin		β-conlycinin	
	Content, mg/g	Degradation^1^, %	Content, mg/g	Degradation, %
SBM 0 h	175.14 ± 4.05^a^	–	135.56 ± 4.10^a^	–
FSBM 24 h^2^	55.80 ± 1.89^b^	68.14	45.54 ± 2.73^b^	66.41
FSBM 48 h^3^	40.95 ± 0.79^c^	76.62	38.27 ± 0.93^c^	71.77
FSBM 72 h^4^	37.48 ± 1.31^c^	78.60	36.75 ± 0.95^c^	72.89

Values are means of three replicates per treatment. Means in a row without common superscript differ significantly (P < 0.05)

^1^Degradation rate = (soybean antigenic protein content in uninoculated SBM − soybean antigenic protein content in FSBM)/soybean antigenic protein content in uninoculated SBM × 100%

^2^ FSBM 24 h: the soybean meal was inoculated with *B. velezensis* 157 and fermented at 37 °C for 24 h

^3^ FSBM 48 h: the first-stage fermentation was inoculated with *B. velezensis* 157, incubated at 37 °C for 24 h, then were inoculated with *L. plantarum* BLCC2-0015 and fermented under anaerobic conditions at 37 °C for another 24 h, totally 48 h

^4^ FSBM 72 h: the first-stage fermentation was inoculated with *B. velezensis* 157, incubated at 37 °C for 24 h, then were inoculated with *L. plantarum* BLCC2-0015 and fermented under anaerobic conditions at 37 °C for another 48 h, totally 72 h

### Chemical composition

The nutrient content characteristics of uninoculated SBM and FSBM samples after two-stage fermentation were presented in Table 2. The crude protein content was 47.28 ± 0.34%. After 24 h of fermentation, the crude protein content increased to 51.08 ± 0.23% and further reached to 52.36 ± 0.58% during 24–48 h fermentation. Finally, the crude protein content decreased to 51.97 ± 0.44% during 48-72 h fermentation (*P* > 0.05). The TCA-SP content increased significantly from 5.07 ± 0.06% to 10.53 ± 0.10% was observed in the following 24 h, and gradually increased to 11.58 ± 0.13% and 11.79 ± 0.13%, respectively, during the 24 h to 48 h and 48 h to 72 h time period. FSBM contained greater concentrations of Ca, ash and total P than uninoculated SBM. However, the content of the samples increased during the 0 h to 48-h time period, and decreased following the 48–72 h. The hemicellulose content decreased significantly from 19.90 ± 0.14% to 14.33 ± 0.09% during the first-stage processes. The relative cellulose and hemicellulose degradation rate of uninoculated SBM were significantly reduced by 22.10% and 39.15% observed during two-stage fermentation, respectively. Notably, inoculation with both *B. velezensis* 157 and *L. plantarum* BLCC2-0015 markedly altered AA composition profiles of uninoculated SBM. All the AA content increased during the 0–24 h period time, then the AA content showed marginal enrichment during the second stage fermentation. Correspondingly, the difference of AA content between the 24 h, 48 h and 72 h of fermentation was not significant (*P* > 0.05). Ultimately, as compared to uninoculated SBM, the total AA content of first-stage fermented feed markedly increased from 41.72 ± 0.40% to 47.14 ± 0.14%, which increased by 1.13 times. Additionally, no significant difference in AA content was observed between first-stage and two-stage fermentation.

**Table 2:** Analyzed nutrient composition of uninoculated SBM and FSBM during two-stage fermentation

Item (%)	SBM 0 h	FSBM 24 h^1^	FSBM 48 h^2^	FSBM 72 h^3^
DM	93.35 ± 0.46^a^	92.79 ± 0.42^ab^	91.84 ± 0.96^bc^	90.88 ± 0.66^c^
CP	47.28 ± 0.34^c^	51.08 ± 0.23^b^	52.36 ± 0.58^a^	51.97 ± 0.44^a^
TCA-SP	5.07 ± 0.06^d^	10.53 ± 0.10^c^	11.58 ± 0.13^b^	11.79 ± 0.13^a^
Crude fibre	5.29 ± 0.08^b^	5.72 ± 0.08^a^	5.40 ± 0.07^b^	5.14 ± 0.05^c^
Cellulose^3^	7.42 ± 0.08^a^	5.95 ± 0.09^b^	5.88 ± 0.09^b^	5.78 ± 0.10^b^
Hemicellulose^4^	19.90 ± 0.14^a^	14.33 ± 0.09^b^	12.59 ± 0.22^c^	12.11 ± 0.15^d^
Ash	6.16 ± 0.06^d^	6.58 ± 0.07^a^	6.38 ± 0.06^b^	6.27 ± 0.03^c^
Ca	0.33 ± 0.01^c^	0.35 ± 0.01^ab^	0.36 ± 0.01^a^	0.34 ± 0.01^bc^
Total P	0.61 ± 0.01^c^	0.71 ± 0.01^a^	0.70 ± 0.01^a^	0.69 ± 0.01^b^
Indispensable AA				
Arg	3.18 ± 0.04	3.25 ± 0.01	3.40 ± 0.23	3.37 ± 0.13
His	1.09 ± 0.02^b^	1.25 ± 0.01^a^	1.25 ± 0.09^a^	1.26 ± 0.06^a^
Ile	1.99 ± 0.05	2.24 ± 0.01	2.19 ± 0.14	2.20 ± 0.11
Leu	3.62 ± 0.04	4.03 ± 0.03	3.95 ± 0.29	3.95 ± 0.19
Lys	2.54 ± 0.02	2.78 ± 0.01	2.72 ± 0.18	2.72 ± 0.13
Met	0.265 ± 0.01	0.32 ± 0.03	0.35 ± 0.04	0.31 ± 0.04
Phe	2.09 ± 0.02	2.42 ± 0.02	2.36 ± 0.23	2.39 ± 0.11
Thr	1.76 ± 0.03^b^	2.00 ± 0.01^a^	2.01 ± 0.06^a^	2.01 ± 0.01^a^
Val	2.14 ± 0.07	2.41 ± 0.03	2.38 ± 0.16	2.36 ± 0.11
Dispensable AA				
Asp	5.14 ± 0.01^b^	5.69 ± 0.03^a^	5.59 ± 0.35^ab^	5.72 ± 0.07^a^
Ser	2.17 ± 0.02^b^	2.45 ± 0.01^a^	2.40 ± 0.16^ab^	2.39 ± 0.09^ab^
Glu	7.89 ± 0.01^b^	9.53 ± 0.06^a^	9.07 ± 0.56^a^	9.05 ± 0.33^a^
Gly	1.94 ± 0.04^b^	2.19 ± 0.01^a^	2.16 ± 0.14^a^	2.22 ± 0.01^a^
Ala	1.98 ± 0.04	2.23 ± 0.02	2.22 ± 0.16	2.24 ± 0.10
Cys	0.41 ± 0.01^b^	0.53 ± 0.01^a^	0.49 ± 0.06^ab^	0.50 ± 0.06^ab^
Tyr	1.21 ± 0.02^b^	1.38 ± 0.01^ab^	1.40 ± 0.13^ab^	1.42 ± 0.07^a^
Pro	2.33 ± 0.03^b^	2.47 ± 0.02^ab^	2.54 ± 0.24^ab^	2.69 ± 0.01^a^
Total AA	41.72 ± 0.40^b^	47.14 ± 0.14^a^	46.47 ± 3.20^ab^	46.77 ± 1.67^ab^

Values are means of three replicates per treatment. Means in a row without common superscript differ significantly (P < 0.05)

^1^ FSBM 24 h: the soybean meal was inoculated with *B. velezensis* 157 and fermented at 37 °C for 24 h

^2^ FSBM 48 h: the first-stage fermentation was inoculated with *B. velezensis* 157, incubated at 37 °C for 24 h, then were inoculated with *L. plantarum* BLCC2-0015 and fermented under anaerobic conditions at 37 °C for another 24 h

^3^ FSBM 72 h: the first-stage fermentation was inoculated with *B. velezensis* 157, incubated at 37 °C for 24 h, then were inoculated with *L. plantarum* BLCC2-0015 and fermented under anaerobic conditions at 37 °C for another 48 h

^4^ Cellulose = ADF − residue after 72% sulfuric acid treatment

^5^ Hemicellulose = NDF − ADF

### Microscopic observation

**Fig. 4:**
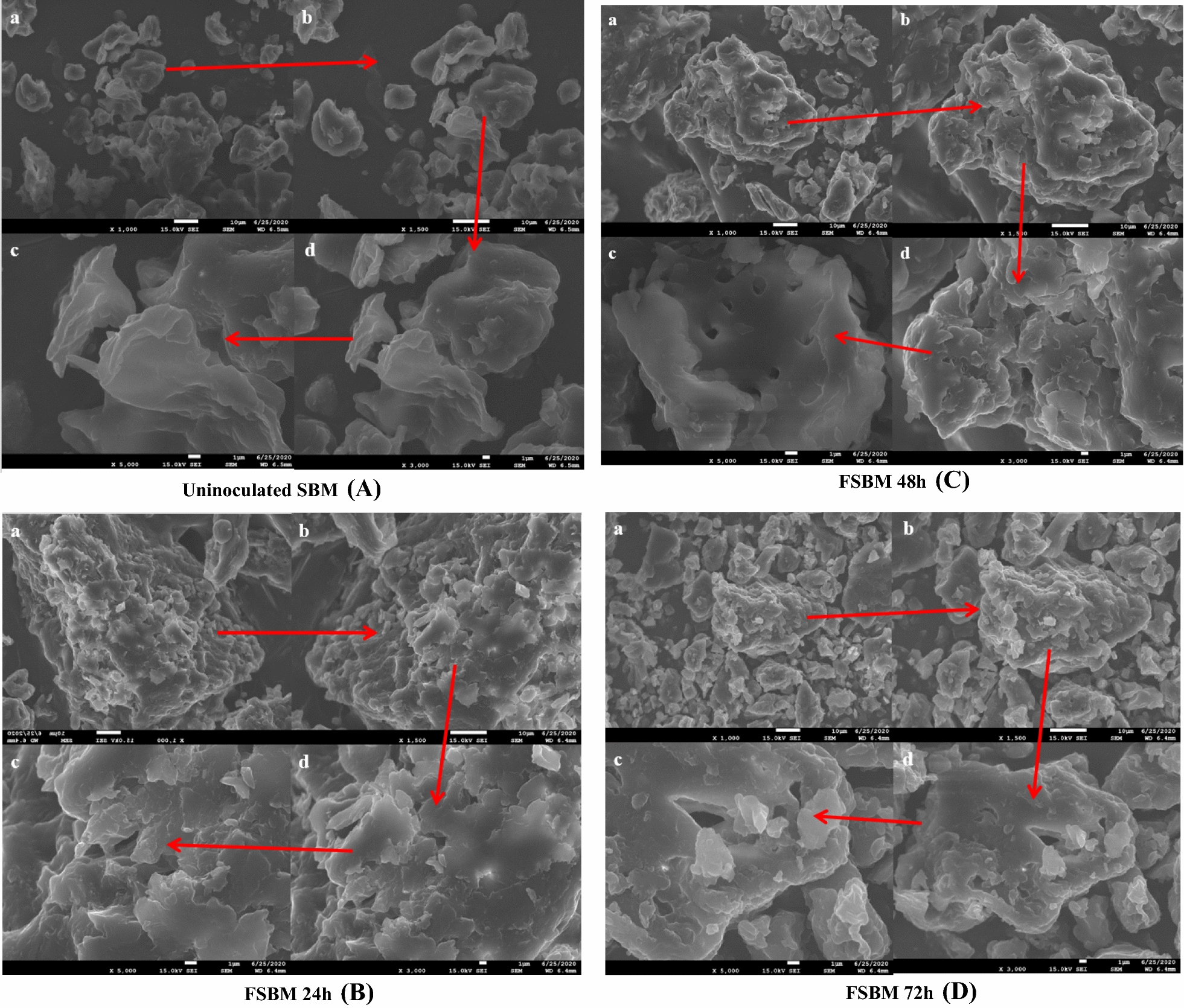
SEM image of uninoculated SBM and FSBM during two-stage fermentation. **a** Uninoculated SBM; **b** the SBM was inoculated with *B. velezensis* 157 and fermented at 37 °C for 24 h; **c** the first-stage fermentation was inoculated with *B. velezensis* 157, incubated at 37 °C for 24 h, then were inoculated with *L. plantarum* BLCC2-0015 and fermented under anaerobic conditions at 37 °C for another 24 h; **d** the first-stage fermentation was inoculated with *B. velezensis* 157, incubated at 37 °C for 24 h, then were inoculated with *L. plantarum* BLCC2-0015 and fermented under anaerobic conditions at 37 °C for another 48 h. All the images were at 1000 (**a**), 1500 (**b**), 3000 (**c**) and 5000 (**d**) fold magnification

The physical structures of uninoculated SBM and FSBM during two-stage fermentation were investigated by SEM. Figure 4 shows the surface images of uninoculated SBM at magnification of ×1000, ×1500, ×3000 and ×5000, with compact and smooth-faced structures. After two-stage fermentation, FSBM showed fragmental, cracked and plurilocellate structures.

## Discussion

Soybean meal production is widespread and abundant, prompting efforts to utilize SBM as an important plant protein resource in animal feed. However, SBM contains several ANFs that limit its extensive use in the diet of young animals (Wongputtisin et al. 2012). Fortunately, several studies have shown that microbial fermentation can degrade, and thus remove, antigenic proteins from SBM to improve its nutritional characteristics (Medeiros et al. 2018; Zheng et al. 2017; Zhu et al. 2017). In this work, a novel two-stage solid-state fermentative system for SBM processing was used to alter the fermentation quality. The contents of glycinin and β-conglycinin in FSBM were degraded by 68.14% and 66.41% during the first fermentation. *B. velezensis* 157 was screened and verified to degrade soybean antigenic protein on a specific screening plate. Meanwhile, many genes encoding proteolytic enzymes of *B. velezensis* 157, such as serine proteases, aminopeptidases, and metalloproteases, were detected in the genome of this strain (Chen et al. 2018b). Correspondingly, the enzyme activity of neutral protease was significantly increased during *B. velezensis* 157 fermentation. Therefore, the hydrolytic effects of the proteases secreted by *B. velezensis* 157 were able to decompose antigenic protein in FSBM during the first fermentation. Aligning with previous results obtained by Shi et al. demonstrating that inoculation of corn-soybean meal mixed feed with *B.subtilis* and *E.faecium* led to degradation of ANFs and enhanced nutritional value. In that study, ELISA analysis revealed glycinin and β-conglycinin degradation rates of 86.12% and 77.53%, respectively, although no soybean antigenic protein degradation occurred during the second fermentative stage (Shi et al. 2017a). Interestingly, in the present study ANFs degradation did occur during the second stage of fermentation, the respective degradation rates of glycinin and β-conglycinin contents further increased to 76.62% and 71.77% during the 48 h fermentation. As the raw materials have been sterilized before fermentation, and the fermentation process is also sterile. No effects of exogenous microorganisms or natural microorganisms present during fermentation are involved. Therefore, *B. velezensis* 157 may still maintain a vital role in the second fermentation, which continues to ferment using residual oxygen in the culture environment. Notably, the results of microbiological counts in this study revealed that *B. velezensis* 157 exhibited the greatest abundance during anaerobic fermentation. Although *L. plantarum* BLCC2-0015 could not degrade soybean antigenic protein as shown in Additional file 1: Figure S1(c), perhaps low pH and structural changes caused by *L. plantarum* BLCC2-0015 may affect the degradation effect of *B. velezensis* 157 as the prolonged fermentation duration. Thus, the content of glycinin and β-conglycinin on FSBM was not further degraded during the process of 72 h fermentation. The protein profile obtained by SDS-PAGE was in accord with the degradation trends of glycinin and β-conglycinin by ELISA analysis during the two-stage fermentation. Moreover, further research is needed to identify the proteins in the fermented products by 2DE and proteomics analysis.

Nevertheless, in this work, FSBM contained greater concentrations of CP and AA content than did uninoculated SBM, with significant differences in amino acid composition after *B. velezensis* 157 treatment, which is consistent with previous experimental research on FSBM (Feng et al. 2007; Frias et al. 2008). It was consistent with reports that the loss of DM may cause the increase of CP and AA during fermentation (Shi et al. 2017b). However, the CP content of FSBM during second-stage fermentation with *L. plantarum* BLCC2-0015 was not changed much. This may be attributed to *L. plantarum* may convert carbohydrate into organic acid. Meanwhile, first-stage fermentation with *B. velezensis* 157 led to an increased TCA-SP level (10.53 ± 0.10%) as compared to the corresponding level in uninoculated SBM, with a slowly increasing observed for second-stage fermentation by *L. plantarum* BLCC2-0015 (11.79 ± 0.125% for fermented SBM vs. 5.07 ± 0.057% for uninoculated SBM). This result is mainly due to hydrolysis of antigenic proteins to form small molecular peptides and free amino acids during fermentation (Gilbert et al. 2008). As an alternative explanation, the increase of TCA-SP might also be due to the hydrolysis of other SBM proteins during two-stage fermentation. During second-stage fermentation, after *L. plantarum* BLCC2-0015 inoculation the pH gradually decreased from 7.02 ± 0.05 to 4.78 ± 0.04, and the lactic acid production was linearly increased. As several previously published studies fermented product with bacterial metabolites, such as acidic substances or bacteriocins, may inhibit proliferation of pathogens (e.g. *Enterobacteriaceae*) (Wang et al. 2015). In this study, FSBM had an antimicrobial effect on the growth of *S. aureus* and *E. coli* in vitro. It had been demonstrated that *B. velezensis* 157 antibacterial effect against a broad spectrum of food-borne pathogens in our previous study (Chen et al. 2018b). Additionally, the high LA content and low pH of FSBM during second fermentation are essential for pathogen inhibition (Zhu et al. 2017). Thus, The FSBM showed potential for compensate the use of antibiotics in feed.

In the present study, levels of hemicellulose and cellulose in uninoculated SBM declined by 27.99% and 19.81%, respectively, and were most significantly altered during first-stage fermentation in the presence of *B. velezensis* 157, after which hemicellulose levels continued to decrease, reaching a lower level of 39.15% during second-stage fermentation. Moreover, several NSP-degrading enzymes activities (NSPases, cellulase, xylanase, β-mannanase, and pectinase) were determined from the fermented product during the two-stage fermentation, which may cause the breakdown of antinutritional substrates above. Additionally, With the degradation of cellulose and hemicellulose, the protein would be degraded easily by protease of *B. velezensis* 157. Correspondingly, it was observed by SEM that the surface structure of FSBM showed cracked and porous structure compared with uninoculated SBM after fermentation. This indicated that the lignocellulose component of SBM may be degraded and such changes of surface structure in FSBM may drive by NSPases and protease secreted during the process. Therefore, lower cellulose and hemicellulose indicated that FSBM may have higher nutrient digestibility than the uninoculated SBM. Furthermore, comparative genome analysis of *B. velezensis* has detected genes encoding common lignocellulolytic enzymes that could effectively degrade cellulose and hemicellulose, but failed to identify a cellobiohydrolase that could directly hydrolyze cellulose (Chen et al. 2018a), explaining the limited cellulose degradation rate observed here during two-stage fermentation. By contrast, the hemicellulose degradation rate approached 39% during two-stage fermentation, a result likely since *B. velezensis* 157 is equipped with numerous hemicellulose-degrading enzymes, such as 1,4-β-xylosidase, endo-1,4-β-xylanase, arabinan endo-1,5-α-l-arabinosidase, α-*N*-arabinofuranosidase, arabinoxylan arabinofuranohydrolase, β-mannosidase, arabinogalactan endo-1,4-β-galactosidase, and glucuronoxylanase. Moreover, carbohydrate esterases, pectate lyase, and carbohydrate-binding modules (CBMs) that are known to be produced by *B. velezensis* 157 may also influence cellulose and hemicellulose degradation. However, to date few published reports have described the effects of these novel lignocellulolytic enzymes on lignocellulose biodegradation aside from enzymes produced by *B. velezensis* strains ZY-1-1 (Zhang et al. 2018) and ASN1 (Nair et al. 2018). Notably, recent whole-genome analysis reports of *B. velezensis* strains GH1-13 (Kim et al. 2017), FZB42 (Fan et al. 2018), UCMB5113 (Niazi et al. 2014), and LS69 (Liu et al. 2017) predict a series of lignocellulose -degrading enzyme genes that may play a role in bacterial colonization of plant roots. Nevertheless, little research has been published describing *B. velezensis* as an animal feed probiotic, prompting us to optimize its use in fermentation to remove ANFs from SBM for livestock feed use.

In summary, during two-stage SBM fermentation induced by stepwise inoculation of *B. velezensis* 157 followed by *L. plantarum* BLCC2-0015. The results indicated that *B. velezensis* 157 could significantly alter the nutritional characteristics of uninoculated SBM based on degrading ANFs (soybean antigenic protein, cellulose, and hemicellulose), changing physicochemical features and functional metabolites, while *L. plantarum* BLCC2-0015 mainly functioned to reduce pH and generate high lactic acid concentration for pathogen inhibition and feed preservation. Therefore, *B. velezensis* 157 holds great promise as an animal feed additive that enhances agricultural products’ value.

## Supplementary information

**Additional file 1: Figure S1.** Protein degradation capacity of B. velezensis 157 and L. plantarum BLCC2-0015.** Figure S2.** Antimicrobial activity of the fermented product
