# Supplementary material for: Nutritional quality improvement of soybean meal by Bacillus velezensis and Lactobacillus plantarum during two-stage solid- state fermentation
Source: AMB Express. 2021 Feb 5;11:23. doi: 10.1186/s13568-021-01184-x (PMC7865068; doi:10.1186/s13568-021-01184-x)
Supplement: Supplementary file 1 — Additional file 1: Figure S1. Protein degradation capacity of B. velezensis 157 and L. plantarum BLCC2-0015. Figure S2. Antimicrobial activity of the fermented product [file 13568_2021_1184_MOESM1_ESM.pdf]

AMB Express

Nutritional quality improvement of soybean meal by *Bacillus velezensis* and *Lactobacillus plantarum* during two-stage solid- state fermentation

Long Chen[1], Zijian Zhao[2], Wei Yu[1], Lin Zheng[1], Lijia Li, Wei Gu[3], Haiyan Xu[3], Bingdong Wei[1*], Xiaogang Yan[1*]

Institutions:

1 Branch of Animal Husbandry, Jilin Academy of Agricultural Sciences, Jilin Gongzhuling 136100, China;

2 Institute of Agro-food Technology, Jilin Academy of Agricultural Sciences, Changchun, 130033, China;

3 Shandong BaoLai-LeeLai Bioengineering Co. Ltd., Tai'an 271000, Shandong, People's Republic of China

Corresponding authors:

Bingdong Wei

Branch of Animal Husbandry, Jilin Academy of Agricultural Sciences, No. 186 Dong xinghua Street, Gongzhuling, Jilin Province, 136100, P.R. China

Tel: +86-0434-5163761;

Fax: +86-0434-5163761

E-mail: weibingdong@dlut.edu.cn

Xiaogang Yan

Branch of Animal Husbandry, Jilin Academy of Agricultural Sciences, No. 186 Dong xinghua Street, Gongzhuling, Jilin Province, 136100, P.R. China

Tel: +86-0434-5163753;

Fax: +86-0434-5163753

E-mail: yanxiaogang1977@163.com

Supplemenary:

Figure S1. The protein degradation capacity of *B. velezensis* 157 (a) and *L. plantarum* BLCC2-0015(c), liquid LB (c) and MRS (d) medium were used as the control group.

Figure S2. Assessment of antimicrobial activity of the fermented product. (A-C) Antimicrobial activity of the FSBM (1-3) against *Staphylococcus aureus* ATCC25923 compared with uninoculated SBM (control). (D-F) Antimicrobial activity of the FSBM (1-3) against *Escherichia coli* ATCC25922 compared with uninoculated SBM (control). 1, 2 and 3 represent the antimicrobial results from three independent fermentation experiments.

B. velezensis 157

L. plantarum BLCC2-0015

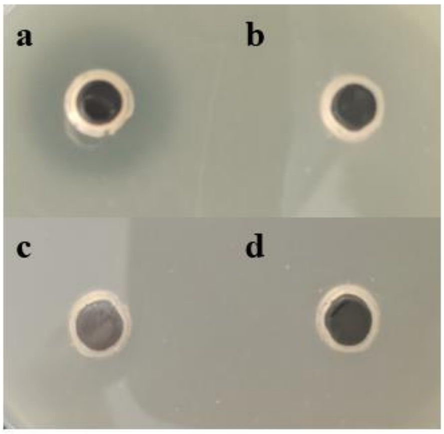

Figure S1

Staphylococcus aureus
ATCC25923

Escherichia coli
ATCC25922

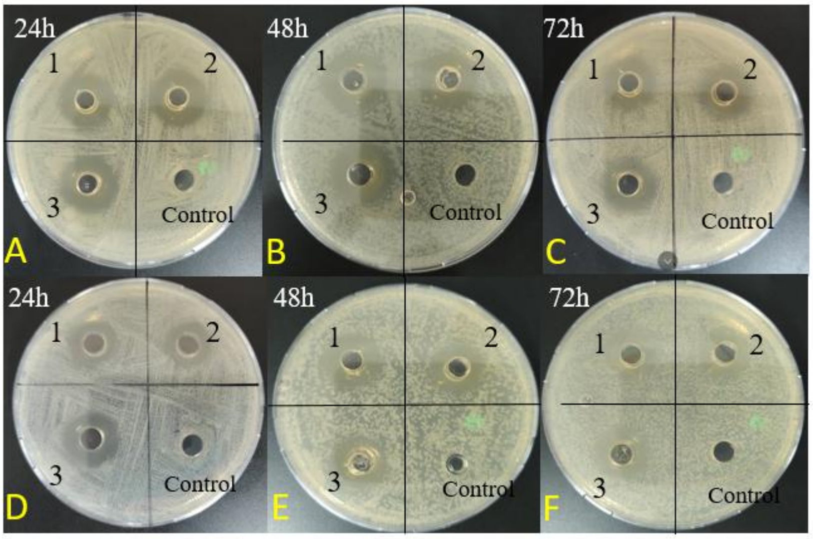

Figure S2
